# Prevention and management of chemotherapy-induced diarrhea in patients with colorectal cancer: a consensus statement by the Canadian Working Group on Chemotherapy-Induced Diarrhea

**DOI:** 10.3747/co.2007.96

**Published:** 2007-02

**Authors:** J.A. Maroun, L.B. Anthony, N. Blais, R. Burkes, S.D. Dowden, G. Dranitsaris, B. Samson, A. Shah, M.P. Thirlwell, M.D. Vincent, R. Wong

**Affiliations:** * Ottawa Hospital Regional Cancer Centre, Ottawa, Ontario; † University Hospital, lsuhsc New Orleans, New Orleans, Louisiana, U.S.A.; ‡ Notre-Dame Hospital, chum, Montreal, Quebec.; § Mount Sinai Hospital, Princess Margaret Hospital/uhn, Toronto, Ontario.; || Tom Baker Cancer Centre, Calgary, Alberta.; # Outcomes research consultant, Toronto, Ontario; ** Hôpital Charles-Lemoyne, Greenfield Park, Quebec; †† BC Cancer Agency, University of British Columbia, Vancouver, British Columbia.; ‡‡ Montreal General Hospital, Montreal, Quebec.; §§ London Health Sciences Centre, London, Ontario.; |||| St. Boniface General Hospital, Winnipeg, Manitoba

**Keywords:** Chemotherapy, diarrhea, cid, octreotide, 5-fu, irinotecan, prophylaxis, management

## Abstract

Chemotherapy-induced diarrhea (cid) is a common side effect of cancer treatment and can cause significant morbidity and mortality. Diarrhea is frequently severe enough to require a dose reduction of, a delay in, or a discontinuation of chemotherapy. Diarrhea-associated mortality has been reported to be as high as 3.5% in clinical trials of irinotecan and bolus 5-fluorouracil in colorectal cancer. The frequency of cid and its impact on patient management are frequently under-recognized in clinical practice.

A Canadian working group, consisting of medical oncologists and an oncology pharmacist, was formed in 2001 to review the optimal approach to managing cid and to identify and implement new areas of research. The recommendations that follow are the result of the group’s work.

Acute medical management of cid includes loperamide or diphenoxylate as first-line agents. Subcutaneous octreotide is recommended for intractable grade 2 diarrhea and may be considered for grade 1 cid that does not resolve with high-dose loperamide. Hospitalization is recommended for patients with grades 3 and 4 cid; in-hospital care includes rehydration, antibiotic therapy, and octreotide.

A chemotherapy dose reduction is generally advised for patients who have experienced grade 3 or 4 diarrhea in a previous chemotherapy cycle. If a dose reduction is not desired, prophylaxis with intramuscular long-acting release octreotide may be considered.

The foregoing recommendations are based on expert opinion and require validation in prospective clinical trials.

## 1. INTRODUCTION

Diarrhea is a common side effect of chemotherapy, notably those regimens that include bolus fluorouracil (5-fu) and irinotecan. The incidence of all grades of diarrhea during chemotherapy has been reported to be as high as 82%, with up to one third of patients experiencing severe (grade 3 or 4) diarrhea (Table I)^2^,^18^.

The frequency of chemotherapy-induced diarrhea (cid) is well-established, but its impact on patient morbidity and mortality may be under-recognized. Chemotherapy-induced diarrhea can be severe enough to result in fluid and electrolyte losses, which can cause potentially life-threatening dehydration, electrolyte imbalances, and renal insufficiency; in nutritional deficiencies from alterations in gastrointestinal (gi) transit and digestion; and in adverse effects on quality of life. Diarrhea is also associated with an increased risk of infectious complications in patients with chemotherapy-induced neutropenia.

The onset of cid may also require that the patient’s dose of the chemotherapy regimen be reduced or discontinued, with possible adverse effects on patient outcome, particularly in the curative adjuvant setting. In a retrospective analysis of 100 consecutively treated colorectal cancer patients with diarrhea, 45% of the 673 chemotherapy cycles were associated with cid. Overall, 52 of 100 patients experienced severe diarrhea (grade 3 or 4), and 56 patients required a modification in chemotherapeutic regimen (dose reduction, delay in therapy, or discontinuation) 19. One third of patients in that series required treatment other than oral antidiarrheal medications, 23% required hospitalization, and 21% received intravenous fluids. Similarly, Petrelli *et al.* 20,21 reported that more than 40% of subjects on a regimen of 5-fu with high-dose leucovorin (lv) developed diarrhea, and 52% of those patients required a reduction of their chemotherapy dose.

A Canadian Working Group retrospective analysis of 63 colorectal cancer patients requiring hospitalization after receiving a variety of chemotherapy regimens with or without concurrent radiotherapy found that 58% developed severe diarrhea after the first cycle of chemotherapy and that 59.6% required a reduction of, change in, or discontinuation of their chemotherapy regimen (Table II)^22^. An analysis of two trials sponsored by the U.S. National Cancer Institute (nci) reported that cid may be associated with a substantially increased risk of early mortality among patients treated with the Saltz regimen 23 of irinotecan/bolus 5-fu/lv (ifl) 24. In the North Central Cancer Treatment Group trial N9741, involving patients with advanced metastatic colorectal cancer, the rate of treatment-induced or treatment-exacerbated mortality with the ifl regimen (10 of 289 patients—3.5%) was three times that seen with the oxaliplatin plus 5-fu/lv (3 of 277 patients—1.1%) or oxaliplatin plus irinotecan (3 of 277—1.1%) regimens.

In the Cancer and Leukemia Group B adjuvant treatment trial C89803, mortality was 2.5% with the ifl regimen and 0.8% with bolus weekly 5-fu/lv. An independent review panel concluded that most of the deaths could be attributed to gi toxicity and cardiovascular events 24. The gi toxicity deaths were attributable mostly to a syndrome comprising severe diarrhea, nausea and vomiting, anorexia, and abdominal cramping. Associated features included severe dehydration, neutropenia, fever, and electrolyte imbalances.

A further consideration is the cost of cid management. In a retrospective analysis by Dranitsaris *et al.* 22 of a Canadian cohort of patients with colorectal cancer who had undergone chemotherapy and required hospital admission after the occurrence of grade 3 or 4 cid, the median stay was 8 days. Of 63 patients, 55 (87.3%) required parenteral support, and 50 (79.3%) received intravenous or oral antibiotics. There were 9 deaths recorded (14.3%). The median time to resolution of cid was 12 days. The estimated cost impact of cid was CA$8230 per patient, of which CA$6314 (76.7%) was for hospitalization. That cost exceeds the estimated cost of other toxic events associated with chemotherapy that require hospitalization, such as the cost of febrile neutropenia (CA$5871) and cardiotoxicity (CA$4626) in breast cancer patients (2004 Canadian dollars) 25.

The significant morbidity and mortality associated with cid have demonstrated the need for a more comprehensive assessment of diarrhea and a more aggressive and systematic treatment approach. The Canadian Working Group on Chemotherapy-Induced Diarrhea was formed to address the need for more effective detection and management of cid. The Group’s consensus recommendations on the prevention and management of cid follow. These recommendations expand upon guidelines previously developed by Wadler *et al.* 26,27 and by Cancer Care Ontario 28, and they address the potential use of antidiarrheal prophylaxis in high-risk patients. Where level 1 evidence was lacking, the recommendations were based on expert clinical opinion.

## 2. CONSENSUS STATEMENT

### 2.1 Grading of CID

#### Recommendation

The severity of cid should be evaluated using the current nci criteria (Table III) 29. However, because the nci criteria do not provide a complete assessment of diarrhea, additional information (duration of the diarrhea episode, stool characteristics, and coexisting symptoms) should be obtained during the patient evaluation. In view of the importance of cid in many modern chemotherapeutic regimens used in colorectal cancer, it might be appropriate to modify the nci criteria, taking the above points in consideration, to provide a more relevant diarrhea grading.

### 2.2 Identification of CID Risk Factors

Few studies have attempted to identify risk factors for the development of cid. Cascinu *et al.* 30 reported that the presence of a primary tumour, past history of cid, and chemotherapy administered during the summer months were possible risk factors. Other studies have indicated that patient age (older > younger) and sex (female > male) are additional risk factors 31,32.

The Canadian Working Group is currently conducting a retrospective multicentre case analysis of 319 patients with colorectal cancer. According to the preliminary multivariate analysis, resection of the primary tumour in the bowel and irinotecan chemotherapy are significant risk factors for cid 33. Patients treated in an adjuvant (as opposed to metastatic) setting are also at greater risk of severe cid.

The Working Group is using these data to develop a model for predicting the patients that are at greater risk of severe diarrhea 33. Important variables in the predictive index include the first cycle of chemotherapy (as opposed to later cycles, during which the cid risk declines), a cycle duration greater than 3 weeks, coexisting neutropenia, and the presence of concomitant symptoms (stomatitis, emesis, anorexia, anemia, cramps, or a combination of these). The development of grade 1 or 2 cid following the first cycle was seen as protective for future severe events—likely because of the initiation of more intensive early monitoring.

#### 2.2.1 Future Directions

The preliminary model requires external validation before a predictive index can be implemented in clinical practice. Nevertheless, a predictive model would be invaluable in optimizing cancer therapy.

In addition to the clinical assessment of risk factors, genetic changes at the molecular level might be evaluated to identify the risk of toxicities with chemotherapeutic agents. The two main examples of interest in the treatment of colorectal cancer relate to 5-fu and irinotecan:

- Dihydropyrimidine dehydrogenase (dpd) plays a central role in 5-fu metabolism, and dpd deficiency is well documented as possibly resulting in severe 5-fu–associated toxicity 34.
- The morbidity associated with irinotecan chemotherapy may also be reduced by screening candidates for uridine diphosphate glucuronyl transferase (ugt) polymorphisms. Polymorphisms in this enzyme affect glucuronidation of the irinotecan metabolite sn-38. One study reported that the incidence of grades 3 and 4 diarrhea is 70% in subjects with seven repeats (TA_7_) in the *UGT1A1* isoenzyme promoter region, as compared with 15% in patients with normal alleles 35. The TA_7_ genotype is also associated with an increased risk of neutropenia 35,36. While not routinely performed at present, testing for dpd levels and *UGT1A1* may soon become more widely available for patients receiving 5-fu or irinotecan. The U.S. package insert for irinotecan now recommends a lower starting dose for patients known to be homozygous for the UGT1A1*28 polymorphism 37.

### 2.3 Investigations

#### Recommendation

Patients should be evaluated for possible causes of diarrhea such as medications (for example, laxatives, stool softeners, antacids, antibiotics) and diet (for example, fibre, lactose), comor-bid infection, partial intestinal obstruction or fecal impaction, surgery (for example, post-gastrectomy, short-bowel syndrome), and acute or chronic radiation toxicity.

#### Recommendation

The recommended laboratory investigations include a complete blood count and differential to rule out neutropenia; blood chemistry to determine the presence of electrolyte abnormalities and to assess renal function; and stool analyses in cases of persistent diarrhea to identify possible bacterial (*Clostridium difficile, C. perfringens, Salmonella* species, *Shigella* species), fungal (*Candida*), parasitic (*Giardia*) or viral (rotavirus) pathogens. Other investigations may be warranted, such as abdominal X-ray to rule out coexisting disorders (bowel obstruction, perforation) and endoscopy or biopsy where indicated.

### 2.4 Patient Management: Acute Setting

The development of cid requires prompt, effective intervention. The general management of cid may include bowel rest, hydration, and replacement of electrolytes. Hospitalization is required for patients with dehydration, fever, neutropenia, or nausea and vomiting that prevents adequate oral hydration.

Patients should be advised to modify their diet to eliminate substances that may contribute to diarrhea (for example, milk and high-fat foods) and should be instructed to avoid caffeinated beverages and alcohol 38. Patients should be advised to increase their intake of oral fluids suitable for rehydration.

**FIGURE 1:**
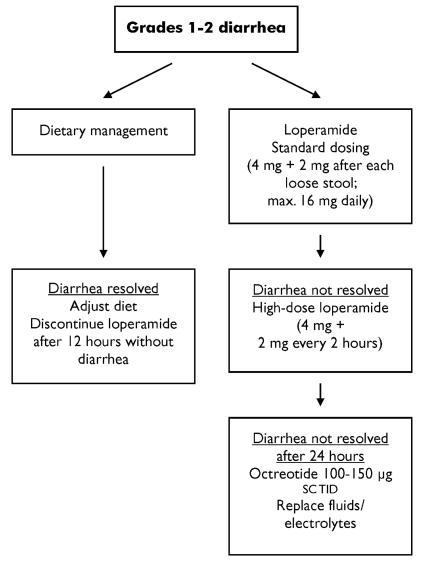
Acute management of grades 1 and 2 diarrhea. sc = subcutaneously; tid = three times daily.

The conventional first-line treatments for cid are the opioids loperamide and diphenoxylate. Double-blind trials indicate that loperamide is the more effective agent of the two for acute diarrhea 39–41. The recommended standard dose of loperamide is 4 mg initially, followed by 2 mg with every episode of diarrhea to a maximum of 16 mg daily 42,43. High-dose loperamide (4 mg initially, followed by 2 mg every 2 hours) appears to be moderately effective in irinotecan-induced diarrhea 44,45. The maximum dose for irinotecan-induced diarrhea is 24 mg daily (Figure 1).

For intractable grade 1 or 2 diarrhea or *de novo* grade 3 or 4 diarrhea, the somatostatin analogue octreotide is the recommended treatment. In clinical trials, octreotide 100–150 μg subcutaneously three times daily has been shown to be effective in resolving grades 3 and 4 diarrhea in 60%–95% of patients after chemotherapy 46–48 or pelvic radiotherapy 49. A higher octreotide dose (500 μg subcutaneously three times daily) has been shown to be well-tolerated 50 and more effective in resolving intractable diarrhea in chemotherapy patients 48. The increased cost of the higher dose may be justified by the reduction in hospitalization time, but this hypothesis requires further study.

#### Recommendation

Patients developing diarrhea should be treated with standard doses of loperamide. A higher dose may be employed for mild-to-moderate diarrhea (grade 1 or 2) that persists for more than 24 hours. Loperamide should be discontinued 12 hours after diarrhea resolves and a normal diet has been re-established.

#### Recommendation

**FIGURE 2:**
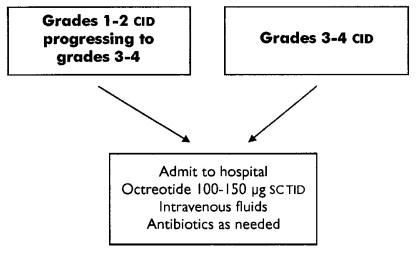
Acute management of grades 3 and 4 diarrhea. cid = chemotherapy-induced diarrhea; sc = subcutaneously; tid = three times daily.

Hospitalization should be considered for patients with grade 2 diarrhea that does not resolve after 24 hours of high-dose loperamide, particularly if they show evidence of clinical deterioration. Hospitalization is recommended for all patients with severe diarrhea (grades 3 and 4; Figure 2). Patients managed in hospital should receive intravenous fluids for correction of dehydration and electrolyte abnormalities as needed, and antibiotics (for example, fluoroquinolone).

#### Recommendation

Grade 2 diarrhea refractory to high-dose loperamide should be treated with octreotide 100–150 μg subcutaneously three times daily. Octreotide may be considered for patients with grade 1 diarrhea that is refractory to high-dose loperamide. Following octreotide initiation, loperamide dosing may be maintained or discontinued.

#### Recommendation

For patients with severe (grades 3 and 4) diarrhea, octreotide 100–150 μg subcutaneously three times daily is the recommended first-line agent. The dose may be escalated if the patient does not respond within 24 hours. Patients should receive intravenous fluids and electrolytes as needed and antibiotics (for example, fluoroquinolone).

Current guidelines recommend increasing the dose of octreotide in 50-μg increments in patients who do not adequately respond 27, but the Working Group recommends dose escalation to octreotide 300–500 μg subcutaneously three times daily until diarrhea resolves 48. This latter recommendation is based on clinical practice rather than on published trial data. Octreotide should be discontinued 24 hours after the diarrhea has resolved and a normal diet has been re-established.

### 2.5 Patient Management: Prophylaxis

Adjuvant therapy for colorectal cancer poses special challenges, because the intent is curative, and a reduction or delay in the chemotherapy regimen because of severe diarrhea may compromise the outcome of treatment.

Current guidelines express the need for diarrhea prophylaxis, but offer no recommendations. In a preliminary study 51, octreotide 150 μg three times daily administered in conjunction with 5-fu/lv in adult cancer patients was not generally effective. Of 10 patients undergoing treatment, 2 experienced dose-limiting diarrhea. Only 3 patients were able to tolerate 6 weekly chemotherapy treatments without a dose reduction or delay.

A long-acting, slow-release (lar) formulation of octreotide was effective in a small case series of 9 patients with colorectal cancer who developed severe refractory diarrhea after fluoropyrimidine or irinotecan chemotherapy 52,53. All these patients had previously failed to respond to antidiarrheal therapy with loperamide or diphenoxylate, or both. Diarrhea resolved with once-monthly intramuscular injections of octreotide lar 30 mg, and no further hospitalizations for cid were required. The optimal dose of octreotide lar has not been established. The Sandostatin lar Trial for the Optimum Prevention of cid is currently evaluating the efficacy of octreotide lar 30 mg as compared with 40 mg in patients with severe cid.

Currently, no completed large randomized controlled trials have addressed the issue of prophylaxis. (Ongoing trials are discussed at the end of this subsection.) The recommendations that follow are interim suggestions based on the above-cited case series and the expert opinion of the Working Group. Further study is warranted.

#### Recommendation

**FIGURE 3:**
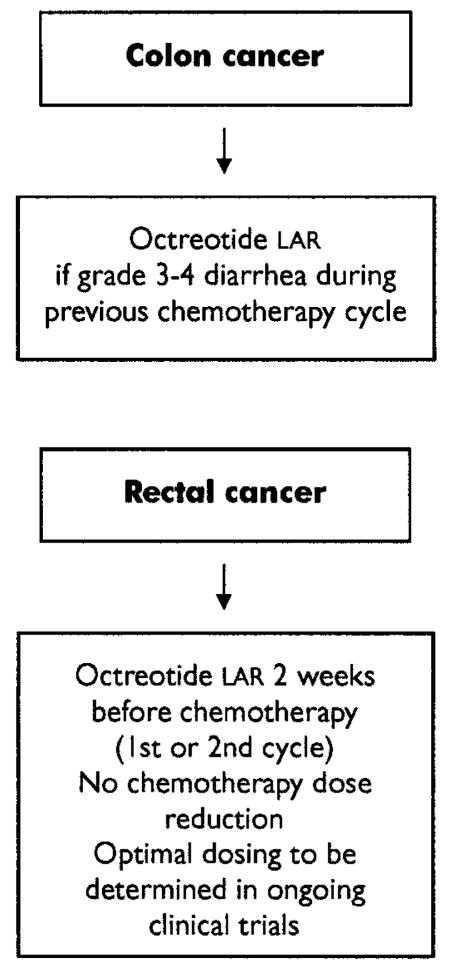
Recommended diarrhea prophylaxis in patients with colorectal cancer treated in an adjuvant setting. lar = long-acting, slow-release formulation.

Octreotide lar 30 mg intramuscularly every 28 days may be considered for patients with colorectal cancer who have experienced grade 3 or 4 diarrhea in a previous chemotherapy cycle and who require another course of chemotherapy, particularly if dose reduction is not desirable (for example, in the adjuvant and neoadjuvant settings; Figure 3).

#### Recommendation

For *de novo* patients with rectal cancer who are undergoing concurrent adjuvant chemotherapy and radiotherapy, no clinical trials have been completed. Octreotide lar 30 mg intramuscularly may be a treatment option in this setting.

The extent of benefit on cid prophylaxis and patient quality of life is not currently known, but is now being investigated in a Canadian multicentre phase iii randomized study of octreotide lar prophylaxis (monthly for 6 months, starting 2 weeks before the first dose of chemotherapy) in patients with rectal cancer treated with postoperative 5-fu and radiation in an adjuvant setting. Octreotide lar is also being evaluated in a phase iii randomized double-blind trial of 226 patients receiving chemoradiotherapy for anal or rectal cancer (rtog-0315) 54. Subjects will receive an octreotide lar or placebo injection 4–7 days before chemoradiotherapy and on day 22 of treatment.

## 3. CONCLUSION

Chemotherapy-induced diarrhea is a severe and frequently undertreated side effect of cancer therapy that requires prompt and effective management to prevent complications, maintain the chemotherapeutic regimen, and improve patient quality of life. A systematic approach to the management of cid is important. Aggressive management with high-dose loperamide or subcutaneous octreotide—and prophylaxis with the intramuscular lar formulation of octreotide—may reduce the morbidity and mortality associated with cid and improve patient outcomes.

Many of the preceding recommendations were perforce based on clinical observation rather than on randomized trials, highlighting the need for more research on the causes of cid and on its optimal management. As part of that effort, the Canadian Working Group is continuing to analyze its colorectal cancer database and available information on genetic changes predisposing to toxicity with chemoradiation regimens to identify possible risk factors, and is completing additional validation work on its predictive model. When the model is complete, the Working Group hopes that it will enable clinicians to identify patients at highest risk of developing cid so that effective and cost-effective prophylactic measures can be instituted. The ongoing Canadian randomized trial of cid prophylaxis in rectal cancer patients receiving adjuvant chemoradiation will provide further information on the role of lar octreotide in cid prophylaxis.

## Figures and Tables

**TABLE I:** Incidence of diarrhea caused by chemotherapeutic agents in colorectal cancer

*Agent*	*Diarrhea grade*	*Incidence (%)*
Intravenous 5-fu + intravenous lv^1^	1 or 2	38
5-fu intravenous push 2	3 or 4	12
5-fu intravenous push + intravenous lv^2^	All	83
	3 or 4	37
5-fu intravenous push + high-dose lv^2^	3 or 4	27
Continuous infusion 5-fu^2^	3 or 4	6
Continuous infusion 5-fu + low-dose lv^2^	3 or 4	11
	3 or 4	11
5-fu 24-hour infusion 2		
Intravenous 5-fu (high dose) + oral lv^3^	3	26
	4	1
Oral tg + oral lv^4^	3	31
Irinotecan intravenous infusion vs. supportive care 5	3 or 4	22
	3 or 4	6
Intravenous paclitaxel 6	3 or 4	17
Continuous infusion 5-fu^7^ vs. intravenous irinotecan 7	3 or 4	11
	3 or 4	22
Intravenous irinotecan 8	All	82
	3 or 4	32
Intravenous irinotecan + intravenous oxaliplatin 9	3 or 4	19
Intravenous irinotecan + intravenous 5-fu + intravenous lv^10^	3 or 4	24
Irinotecan + l-folinic acid + intravenous lv + bolus 5-fu + continuous infusion 5-fu (folfiri) 11,12	3 or 4	11
Oxaliplatin + bolus 5-fu + continuous infusion 5-fu + intravenous lv (folfox) 11,13	3 or 4	11
folfox + bevacizumab 14	3 or 4	11
High-dose continuous infusion 5-fu + intravenous lv + oxaliplatin (flox) 15	All	66
	3 or 4	29
Oxaliplatin + capecitabine + bevacizumab 16	All	73
	3 or 4	30
Irinotecan + bolus 5-fu + lv + bevacizumab 17	3 or 4	32

Adapted, with permission, from *Evaluation and Management Guidelines for the Treatment of Chemotherapy-Induced Diarrhea: A Primer.* Chicago: Robert H. Lurie Comprehensive Cancer Center of Northwestern University; 1999.

5-fu = 5-fluorouracil; lv = leucovorin; tg = tegafur.

**TABLE II:** Clinical and therapeutic sequelae of grades 3 and 4 diarrhea in a retrospective analysis of 63 colorectal cancer patients who were hospitalized for supportive care

*Parameter*	*Prevalence (%)*
Grade 3 or 4 cid	58
Required dose reduction	9.5
Required change in regimen	15.9
Discontinued chemotherapy	34.2
Events associated with cid	
Cramping	39.7
Bloody stools	9.5
Uncontrolled emesis	20.6
Fever requiring antibiotic support	30.2
Stomatitis	7.9
Neutropenia	59.7
Febrile neutropenia	39.7
Mortality	4.8 a
Required additional clinic visit	58.7
Required emergency department visit	42.9

Adapted, with permission, from reference 22.

a Uncontrolled diarrhea may have contributed to 3 of 9 deaths in this series.

cid = chemotherapy-induced diarrhea.

**TABLE III:** National Cancer Institute criteria for severity of chemotherapy-induced diarrhea

*Toxicity grade*	*Criteria*
1	Increase of <4 stools daily over baseline Mild increase in ostomy output as compared with baseline
2	Increase of 4–6 stools daily over baseline Intravenous fluids indicated <24 hours Moderate increase in ostomy output compared with baseline Not interfering with activities of daily living
3	Increase of ≥7 stools daily over baseline Incontinence; intravenous fluids >24 hours Hospitalization Severe increase in ostomy output compared with baseline Interfering with activities of daily living
4	Life-threatening consequences (for example, hemodynamic collapse)
5	Death

Adapted, with permission, from reference 29.
